# Cochlear Implantation Outcome in Children with DFNB1 *locus* Pathogenic Variants

**DOI:** 10.3390/jcm9010228

**Published:** 2020-01-15

**Authors:** Dominika Oziębło, Anita Obrycka, Artur Lorens, Henryk Skarżyński, Monika Ołdak

**Affiliations:** 1 Department of Genetics, Institute of Physiology and Pathology of Hearing, 02-042 Warsaw, Poland; d.ozieblo@ifps.org.pl; 2 Postgraduate School of Molecular Medicine, Medical University of Warsaw, 02-091 Warsaw, Poland; 3 Department of Implants and Auditory Perception, Institute of Physiology and Pathology of Hearing, 02-042 Warsaw, Poland; a.obrycka@ifps.org.pl (A.O.); a.lorens@ifps.org.pl (A.L.); 4 Oto-Rhino-Laryngology Surgery Clinic, Institute of Physiology and Pathology of Hearing, 02-042 Warsaw, Poland; h.skarzynski@ifps.org.pl

**Keywords:** DFNB1 *locus*, hearing loss, deafness, cochlear implants, cochlear outcome, genetics, auditory development

## Abstract

Almost 60% of children with profound prelingual hearing loss (HL) have a genetic determinant of deafness, most frequently two DFNB1 *locus* (*GJB2/GJB6* genes) recessive pathogenic variants. Only few studies combine HL etiology with cochlear implantation (CI) outcome. Patients with profound prelingual HL who received a cochlear implant before 24 months of age and had completed DFNB1 genetic testing were enrolled in the study (n = 196). LittlEARS questionnaire scores were used to assess auditory development. Our data show that children with DFNB1-related HL (n = 149) had good outcome from the CI (6.85, 22.24, and 28 scores at 0, 5, and 9 months post-CI, respectively). A better auditory development was achieved in patients who receive cochlear implants before 12 months of age. Children without residual hearing presented a higher rate of auditory development than children with responses in hearing aids over a wide frequency range prior to CI, but both groups reached a similar level of auditory development after 9 months post-CI. Our data shed light upon the benefits of CI in the homogenous group of patients with HL due to DFNB1 *locus* pathogenic variants and clearly demonstrate that very early CI is the most effective treatment method in this group of patients.

## 1. Introduction

Profound prelingual hearing loss (HL, deafness) occurs before the development of speech and language and represents one of the major congenital defects, which affects as many as 1–6/1000 newborns [1]. Children with profound HL do not develop proper speech, have difficulties in communicating with those around them, and suffer from an impaired intellectual development, which in the long run may lead to social isolation [2], making HL not only a medical but also a social problem. In child development, there is a critical period to acquire language skills and this process is closely related to the presence of auditory stimuli. In children with profound prelingual HL, auditory stimuli can be delivered by a cochlear implant, which compensates for the malfunctioning of the inner ear, directly stimulates the auditory pathway, and enables the processed sound to reach the central nervous system.

Early audiological intervention facilities the development of hearing and speech in children with profound prelingual HL [3]. Cochlear implants offer their users undeniable benefits that have been documented in many studies [2,4,5,6]. However, the excellent outcome of cochlear implantation (CI) does not guarantee that every implanted child will fully benefit in terms of speech understanding and language development. Some cochlear implant users have little or no benefit from using them, and as many as 7% of children stop using cochlear implants [6,7]. The development of auditory competence of cochlear implant users is influenced by many factors, including the etiology of HL, the level of residual hearing preservation, the coexistence of other medical problems, as well as the length of auditory deprivation and the intensity of speech and hearing rehabilitation [8].

Genetic background is the most common cause of congenital HL and it has been estimated that up to 60% of children with profound prelingual HL who receive cochlear implants have an inherited genetic defect leading to deafness. In half of these children, two recessive pathogenic variants are detected in the DFNB1 *locus* (*GJB2/GJB6* genes) [9]. Both genes are expressed in the inner ear (encode connexin 26 and connexin 30, respectively), but the exact mechanism leading to DFNB1-related HL (DFNB1 HL) is still being investigated. Recent data indicate that congenital deafness due to connexin 26 deficiency is primarily associated with cochlear developmental disorders rather than the previously suggested reduction of endocochlear potential and hair cell degeneration. In contrast, mice with double heterozygous connexin 26 and connexin 30 mutations displayed normal cochlear development and their HL is considered to result from reduced endocochlear potential mainly due to impaired heterogenous gap junctional function in the cochlear lateral wall [10,11,12,13].

In this study, we present the first thorough analysis of auditory development in the largest, so far reported, group of cochlear implant recipients with pathogenic variants in the DFNB1 *locus*. As short-term gains in auditory development translate into medium-term gains in social independence and quality of life (presumably through the language competency achieved with a cochlear implant), early auditory development is considered an important outcome measure in children with cochlear implants. In order to relate the CI outcome to genetic background, we decided to carefully select children with cochlear implants to exclude or control the environmental factors leading to HL and affecting auditory development. Additionally, we demonstrate the effect of two factors (auditory responses obtained with hearing aids (HAs) before implantation and the patient’s age at CI) on auditory development of DFNB1 HL children. The available evidence suggests that, in congenitally deaf children, better auditory development is achieved by those who receive cochlear implants earlier rather than later, as well as that CI effectively eliminates differences in auditory development delay observed in patients with a different level of residual hearing.

## 2. Methods

### 2.1. Subjects

A total of 196 prelingually deaf children (89 girls and 107 boys; Table S1) who underwent cochlear implantation between October 2007 and June 2018 at the Institute of Physiology and Pathology of Hearing were recruited for the study based on the following criteria:

- HAs usage prior to CI;
- Cochlear implant received before 24 months of age;
- Completed genetic testing of the DFNB1 *locus;*
- Evaluated auditory development;
- Excluded environmental risk factors for HL development (e.g., severe prematurity, asphyxia, high hyperbilirubinemia, ototoxic drugs, cytomegalovirus infection);
- Excluded risk factors for auditory development (e.g., severe birth defects, deaf-mute parents).

Written informed consent was obtained from the guardians of each participant. The study was approved by the ethics committee at the Institute of Physiology and Pathology of Hearing (IFPS:/KB/01/2012) and performed according to the Declaration of Helsinki.

### 2.2. Genetic Testing

Whole blood or dry blood spot samples were collected from all participating patients. Genomic DNA was isolated with a standard salting-out procedure or using an automatic method (Maxwell RSC Instrument, Promega, Walldorf, Germany), respectively. Genetic testing of the DFNB1 *locus* was performed according to recommendations of the European Molecular Quality Network (EMQN) [14]. In all patients, a tree-step molecular examination procedure was applied. In the first stage, common deletions or insertions in the c.12-72, c.68-198, and c.306-464 regions of the *GJB2* gene (NM_004004.6) were tested using multiplex PCR followed by PCR-restriction fragment length polymorphism (PCR-RFLP) and capillary electrophoresis. In the second stage, allele-specific PCR for the *GJB6* gene (NM_001110219.3) deletions and the real-time PCR genotyping method for the *GJB2* c.-23+1G>A variant were used [15,16]. In the last step, the complete *GJB2* coding region was Sanger sequenced [17].

### 2.3. Audiological Evaluation and Assessment of Auditory Development

All patients recruited for the study were diagnosed with profound prelingual HL and fitted with HAs. The mean age at first HAs fitting was 5.7 months (from 0.9 to 16 months). The mean duration of HAs experience prior to CI was 6.5 months (from 0 to 15.2 months). The audibility provided by HAs was measured as described previously [18]. Maximum audiometric frequencies with children’s behavioral responses were established using developmentally appropriate methods i.e., behavioral observation audiometry, conditioned orientation reflex audiometry, or visual reinforcement audiometry. The tested frequencies included 250, 500, 1000, 2000, and 4000 Hz. On the basis of the results, patients were divided into subgroups with minimal (no free-field responses or responses only up to 500 Hz; n = 100) or wide (free-field responses for at least 250, 500, and 1000 Hz; n = 96) audibility provided by HAs. The mean age of CI was 12.1 months (from 6.9 to 19 months) and patients were classified into subgroups with very early (<12 months of age; n = 111) and early (≥12 months of age; n = 85) CI.

The auditory development of the tested patients was evaluated with the use of the LittlEARS questionnaire (LEAQ) that was previously translated into Polish and validated in groups of normally hearing individuals and patients after CI [18,19,20]. This tool consists of 35 yes/no questions and is dedicated to assess the auditory behavior of children up to 24 months. The auditory development of patients was examined at the time of CI (n = 196) as well as in fifth (n = 196) and ninth (n = 127) month after cochlear implant activation.

### 2.4. Statistical Analysis

The normal distribution of analyzed variables was tested using the Kolmogrov–Smirnow test. A comparison of the two independent groups with normal distribution was performed with a t-Student test, and for groups without normal variable distribution, the Mann–Whitney U test was applied. Multivariable comparisons were conducted using ANOVA. For all analyses, a two-sided *p* value <0.05 was considered statistically significant. Statistical analysis was performed using TIBCO Statistica 13 (Tulsa, OK, USA) and GraphPad Prism 5 (San Diego, CA, USA) software. Scatter plots were generated using the ggplot2 package and R software (R version 3.6.1).

## 3. Results

### 3.1. Genetic Etiology

Genetic testing of the DFNB1 *locus* revealed causal genetic variants in almost 80% (149/196) of subjects. In the majority of them (119/149), the homozygous c.35delG variant was identified. The remaining 30 probands harbored different combinations of variants located in the *GJB2* and *GJB6* genes (Table 1). Among the defective alleles, small deletions, probably resulting in shortening of the protein, were the most frequent. All detected variants were inherited in an autosomal recessive manner. In a group of 47 patients, no pathogenic variants in the *GJB2/GJB6* genes were identified.

### 3.2. Initial Auditory Status of Tested Subjects

**Figure 1:**
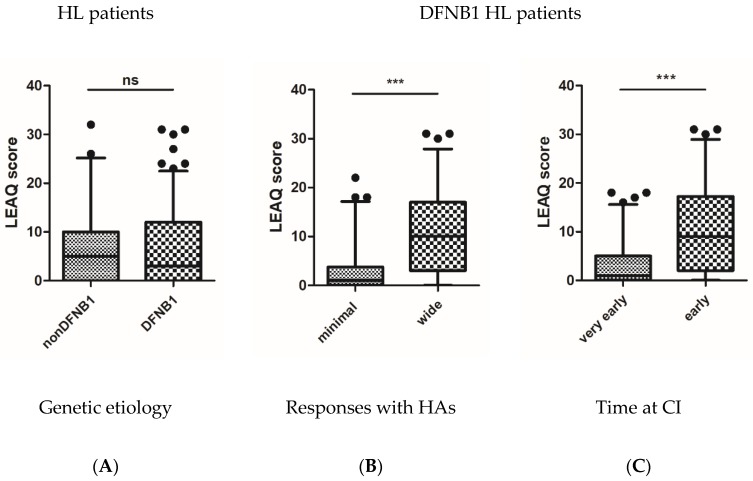
LEAQ scores at the time of cochlear implant activation. (**A**) Differences in LEAQ scores in patients with and without DFNB1 *locus* pathogenic variants; (**B**) differences in LEAQ scores in patients with minimal and wide responses provided by HAs; (**C**) differences in LEAQ scores in patients with very early and early CI. Whiskers represent 5–95 percentile, and black dots indicate outliers. Asterisks represent statistical significance, ***p < 0.001; ns, not significant.

**Figure 2:**
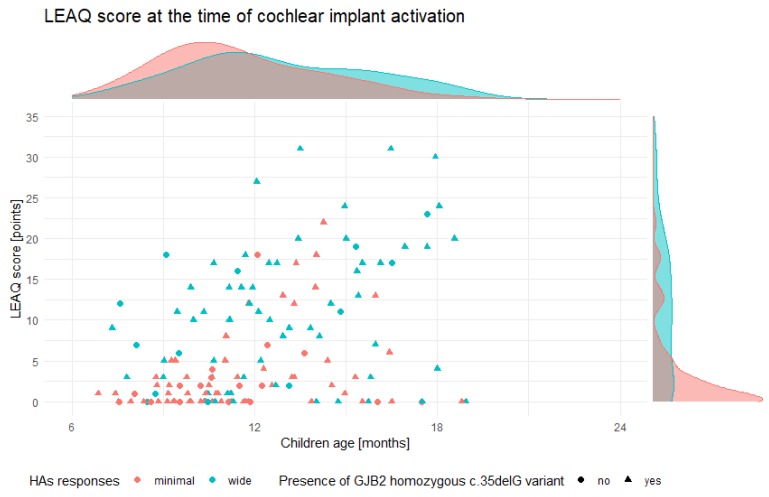
Distribution of LEAQ scores at the time of cochlear implant activation in DFNB1 HL patients.

At the time of cochlear implant activation, mean LEAQ score (0_LEAQ) in the tested children was 6.8 points (min = 0, max = 32; SD = 7.8). There were no statistically significant differences between mean 0_LEAQ score in patients with non-DFNB1 HL and DFNB1 HL (6.85 vs. 6.81; *p* = 0.85) (Figure 1A). After focusing on patients with DFNB1 HL, a variable distribution of 0_LEAQ scores was observed and ranged from 0 to 31 (SD = 7.8). In patients with minimal HAs responses, 0_LEAQ scores cumulated at the bottom of the scale (mean = 3.21, median = 1, mode = 0), while in patients with wide HAs responses, the scores were distributed throughout the scale (mean = 10.56, median = 10, mode = 0) (Figure 2). There were statistically significant differences in mean 0_LEAQ scores between patients with minimal and wide HAs responses (*p* < 0.001) (Figure 1B) as well as between patients implanted very early and early (*p* < 0.001) (Figure 1C). A higher 0_LEAQ score was observed in patients with wide responses in HAs (10.56 vs. 3.21) and later implantation (10.52 vs. 3.87) (Table 2). In the most genetically homogenous group of patients with the *GJB2* homozygous c.35delG pathogenic variant, differences in HAs responses and 0_LEAQ scores were observed (Figure 2). In non-DFNB1 patients, a statistically significant difference in 0_LEAQ was found only for patients implanted very early and early (*p* < 0.05) (Figure S1).

### 3.3. Postoperative Auditory Status of Tested Subjects

**Figure 3:**
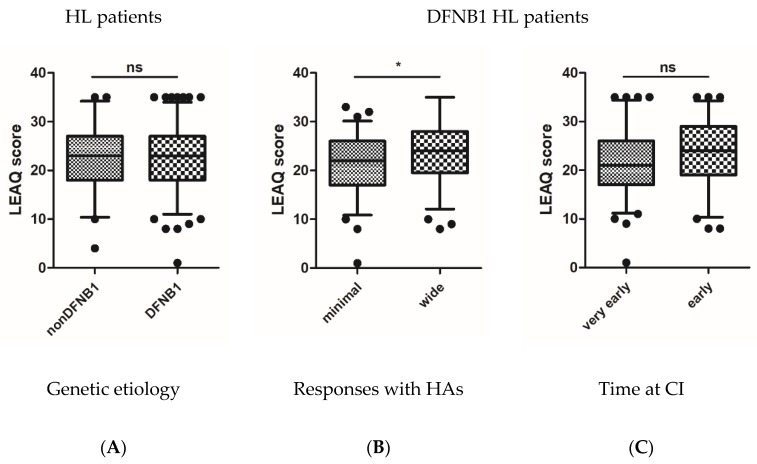
LEAQ scores in the fifth month after cochlear implantation. (**A**) Differences in LEAQ scores in patients with and without DFNB1 *locus* pathogenic variants; (**B**) differences in LEAQ scores in patients with minimal and wide responses provided by HAs; (**C**) differences in LEAQ scores in patients with very early and early CI. Whiskers represent 5–95 percentile and black dots indicate outliers. Asterisks represent statistical significance, * *p* < 0.05; ns, not significant.

**Figure 4:**
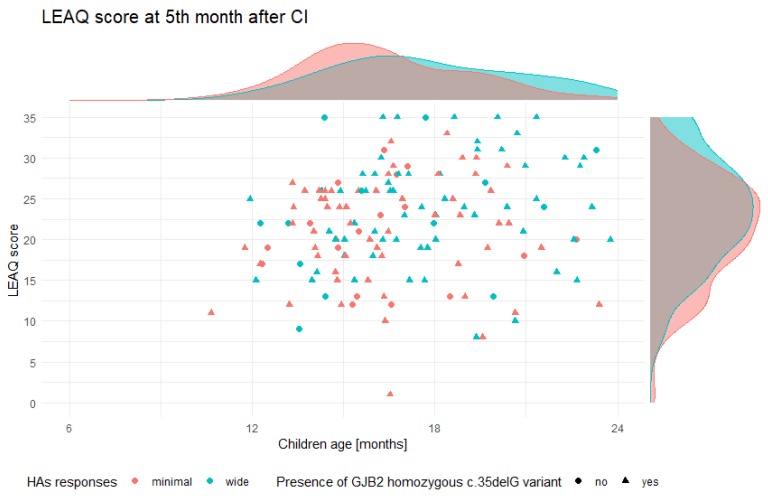
Distribution of LEAQ scores in the fifth month after CI in DFNB1 HL patients.

In the fifth month after CI, the mean LEAQ score (5_LEAQ) in tested children was 22.3 points (min = 1, max = 35; SD = 6.62). There were no statistically significant differences between the mean 5_LEAQ score in patients with DFNB1 HL and non-DFNB1 HL (22.24 vs. 22.47; *p* = 0.84) (Figure 3A). After focusing on patients with DFNB1 HL, a variable distribution of 5_LEAQ scores was observed, ranging from 1 to 35 (SD = 6.7). Both in patients with minimal and wide HAs responses, 5_LEAQ scores had a normal distribution (mean = 20.95, median = 22 vs. mean = 23.59, median = 24) (Figure 4). There was a statistically significant difference in mean 5_LEAQ scores between patients with minimal and wide HAs responses (*p* < 0.05) (Figure 3B), but there was no difference between patients implanted very early and early (*p* = 0.1) (Figure 3C). A better auditory development was still observed in patients with wide responses in HAs (23.59 vs. 20.95) (Table 2). In the most genetically homogenous group of patients, those with the *GJB2* homozygous c.35delG pathogenic variant, differences in 5_LEAQ scores were still observed (Figure 4). In non-DFNB1 patients, no statistically significant differences in the corresponding subgroups in 5_LEAQ were observed (Figure S2).

In the ninth month after CI, there were no statistically significant differences in mean LEAQ score (LEAQ_9) between the studied groups of children. All implanted DFNB1 HL patients reached approximately 28 points (data not shown).

### 3.4. CI Outcomes

**Figure 5:**
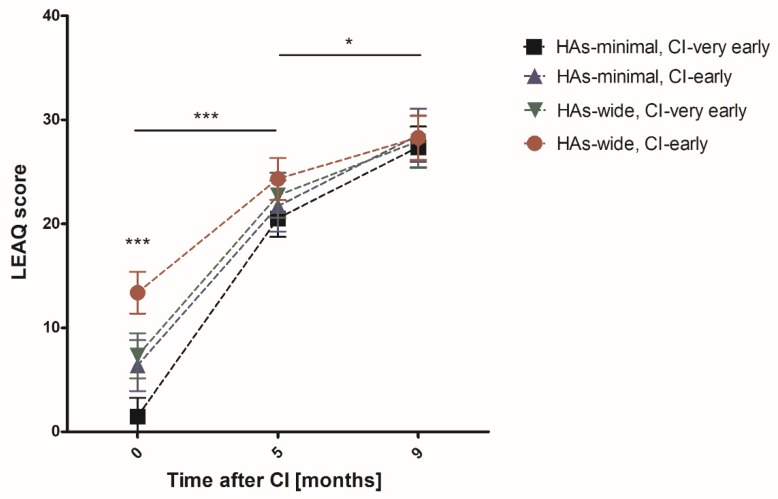
Distribution of an average LEAQ score of patients with DFNB1 HL at subsequent time intervals (mean ± 95% confidence interval). Asterisks indicate statistically significant differences observed between patients with minimal HAs responses and very early CI vs. patients with wide HAs responses and early CI. Asterisks represent statistical significance, * *p* < 0.05, *** *p* < 0.001.

The analysis of auditory development in DFNB1 HL children in all three time intervals revealed statistically significant differences only at the time of CI activation (Figure 5). The highest LEAQ score was observed in patients with wide HAs responses and later implantation (13.38 points). The lowest LEAQ score was presented in the opposite group of children with minimal responses and very early CI (1.47 points) (Table 2). Up to 5 months after CI patients with minimal HAs responses and very early CI reached more LEAQ points than patients with wide HAs and later CI (19.06 vs. 10.95; *p* < 0.001). This strong difference slowly decreased between the fifth and ninth month after CI, and patients with minimal HAs responses and those implanted very early achieved only 2.8 points more than children with wide HAs and later implantation (7.23 vs. 4.43; *p* < 0.05) (Figure 5).

During the analyzed implantation period, no sex-dependent differences were observed.

## 4. Discussion

In this study, we decided to verify the hypothesis of significant auditory development in CI children with DFNB1 *locus* pathogenic variants and analyzed the possible factors determining the CI outcome. For this purpose, we selected a large group of HL patients without environmental factors affecting their hearing and auditory development. Our data clearly show that DFNB1 HL children strongly benefit from CI. Their auditory abilities were growing rapidly and they reached similar levels as in the non-DFNB1 HL group.

### 4.1. Auditory Development of DFNB1 HL Children Receiving CI

Our study on auditory development of CI patients in different time intervals demonstrated that the greatest diversity of LEAQ scores was present at the time of cochlear implant activation. In line with the previously published data, we found that the differences are determined by two independent factors i.e., the patient’s age at CI and auditory responses provided by HAs [18,21]. This difference decreased over time and disappeared in the ninth month after CI. The observation should be interpreted with caution and in relation to LEAQ normative data. As at the ninth month test interval, the children implanted very early were younger than the children implanted early by 4.76 months on average, which in terms of auditory development should be translated into 3.63 LEAQ point differences (older, normally hearing children have more LEAQ scores). In this context, our findings that early and very early implanted children have equal LEAQ scores after 9 months of CI use indicates that very early implanted children have an auditory development closer to that expected in normally hearing children. In this way, we demonstrated that very early CI (before 12 months of age) promotes age appropriate auditory development in the group of CI with DFNB1 *locus* pathogenic variants.

Total LEAQ scores in the children who demonstrated responses over a wide frequency range were significantly higher at activation than in children with minimal responses in HAs. This was due to the fact that these children had already reached a higher level of auditory development with their HAs. This difference was not maintained at 9 months post implantation. Children who had no free-field responses at 0, 5, and 9 months of CI use had a higher rate of auditory development. This indicates that CI can provide effective auditory stimulation and enable children with no HAs responses (no residual hearing) to catch up with those who demonstrated responses over a wide frequency range from HAs prior to implantation (those with residual hearing).

### 4.2. Molecular Mechanism Supporting the Observed CI Outcome

Pathogenic variants at the DFNB1 *locus* (*GJB2* and *GJB6* genes) represent a major genetic determinant of HL in prelingually deaf children. Our results show considerable auditory development in DFNB1 HL patients. It is consistent with (i) the presumed molecular mechanisms leading to the development of DFNB1 HL, (ii) known expression pattern of *GJB2* and *GJB6* genes in the human cochlea [22], and (iii) the spiral ganglion hypothesis. In 2012, Eppsteiner et al. proposed that pathogenic variants in genes having a preferential expression in the cochlea (e.g., *GJB2*, *SLC26A4*, *MYO7A*, *TMC1,* or *COCH*) are associated with a good CI outcome, while mutations in genes expressed in the spiral ganglion (e.g., *TIMM8A*, *OPA1,* or *DIAPH3*), i.e., in the initial parts of the auditory pathway, are associated with a worse CI outcome [23,24,25,26]. Considering the *GJB2* and *GJB6* expression pattern in the auditory system and no data showing dysfunction of spiral ganglion neurons in patients with DFNB1 *locus* pathogenic variants [27,28,29], one should expect good CI performance in this group of patients.

In our study, patients with the DFNB1 *locus* pathogenic variants suffered from profound HL and were qualified for CI. However, even in this homogenous group, we observed differences in the responses obtained with HAs, which is a derivative of a different level of residual hearing in these patients. As patients recruited for the study were rigorously selected, we assume that the observed differences in residual hearing do not arise from the influence of environmental factors, but rather are a consequence of currently unknown genetic modifiers; this phenomenon requires further studies.

### 4.3. Possible Explanations for Uncertain Data from the Literature

Several studies have concentrated on a possible link between genetic etiology of HL and CI outcome [21,25,30], but many of them have provided uncertain and controversial results. Reliable studies assessing this issue require a large number of patients with similar demographic features and a similar influence of environmental factors. Here, we selected a large and homogenous group of CI patients with the DFNB1 *locus* pathogenic variants. The detailed evaluation of their medical records enabled us to exclude major environmental factors affecting hearing and auditory development. All children received a cochlear implant before the 24th month of age and their auditory development was evaluated with LEAQ scores at defined time intervals within one year of cochlear implant activation. All patients were treated in one medical center. Previously published data were based on the analysis of a limited number of patients wherein the most numerous group was almost four times smaller than ours (40 vs. 149) [8,31]. Moreover, there was a large variation in the time of implantation, ranging from 1 to even 14 years of age [8,32]. The patients described in a single study have often been exposed to different environmental factors, such as different lengths of auditory deprivation, as well as having different levels of natural ability to acquire auditory skills [3]. It should also be taken into account that the available questionnaires and objective measurements of CI outcome are usually dedicated to individuals of a particular age, and a direct comparison of the results could be biased and may lead to incorrect conclusions.

### 4.4. Limitations of Our Study and Future Directions

Here, were did not identify any significant differences between DFNB1 and non-DFNB1 patients. This may be due to a possible heterogeneous genetic background in the latter group, its relatively small size, and the wide distribution of LEAQ scores [21,25,31]. We assume that the non-DFNB1 cohort has HL due to molecular defects affecting mainly the cochlea, but we cannot exclude the possibility that individuals with a poor CI outcome may have a molecular defect affecting the neurological component of the auditory system [23,25,26]. From a practical point of view, it is important to dissect the genetic cause of HL in this group of patients. To accomplish this goal, high throughput genetic testing should be implemented. This approach will allow us to identify the causative variants and select a homogeneous group of patients with a poor CI outcome that could serve as an appropriate control group to study CI outcome.

LEAQ is a major tool dedicated to the evaluation of auditory development in infants and toddlers. It is widely used for research purposes, but we are aware of its limitations. CI outcome assessed with the LEAQ score could be biased with the subjectivity resulting from the parent answering the LEAQ questions. One may consider using objective measures such as cortical auditory evoked potentials that depict auditory development with a gradual decrease in P1 latency. However, this method requires elaborated procedures and experienced staff and is more burdensome for young children [33,34].

In this study, we assembled a large and homogenous group of DFNB1 HL patients and performed a detailed analysis of their CI outcome. All patients were implanted before 24 months of age, their hearing behavior was evaluated with LEAQ. Therefore, we were only able to follow their auditory development up to 9 months after CI. Although during this time, we observed the benefits of CI, we cannot predict whether this trend continued in the following months and how the higher auditory functions, in particular speech discrimination, developed. To achieve this goal a longer follow-up period is needed.

## Figures and Tables

**Table 1:** Pathogenic variants of the DFNB1 *locus* detected in tested subjects.

Number of Cases	Allele 1	Allele 2
119	*GJB2* c.35delG	*GJB2* c.35delG
10	*GJB2* c.35delG	*GJB2* c.313_326del
5	*GJB2* c.35delG	*GJB2* c.167delT
3	*GJB2* c.35delG	*GJB2* c.-23+1G>A
3	*GJB2* c.35delG	*GJB6* del(D13S1830)
1	*GJB2* c.35delG	*GJB2* c.102G>A
1	*GJB2* c.35delG	*GJB2* c.109G>A
1	*GJB2* c.35delG	*GJB2* c.235delC
1	*GJB2* c.35delG	*GJB2* c.290insA
1	*GJB2* c.313_326del	*GJB2* c.167delT
1	*GJB2* c.313_326del	*GJB2* c.235delC
1	*GJB2* c.313_326del	*GJB2* c.139G>T
1	*GJB2* c.551G>C	*GJB2* c.551G>C
1	*GJB2* c.-23+1G>A	*GJB6* del(D13S1830)

**Table 2:** LittlEARS questionnaire (LEAQ) scores of the DFNB1 hearing loss (HL) patients in subsequent time intervals.

Analyzed Group	N 0–5	CI Activation	Five Months after CI	N 9	Nine Months after CI
Very early CI	83	3.87 ± 0.57	21.43 ± 0.71	67	27.60 ± 0.59
Early CI	66	10.52 ± 1.11	23.26 ± 0.85	60	28.38 ± 0.76
Minimal HAs	76	3.21 ± 0.58	20.95 ± 0.73	65	27.82 ± 0.64
Wide HAs	73	10.56 ± 0.99	23.59 ± 0.79	62	28.13 ± 0.70
Very early CI; minimal HAs	49	1.47 ± 0.33	20.53 ± 0.88	40	27.38 ± 0.81
Very early CI; wide HAs	34	7.32 ± 1.05	22.74 ± 1.15	27	27.93 ± 0.84
Early CI; minimal HAs	27	6.37 ± 1.31	21.70 ± 1.32	25	28.52 ± 1.07
Early CI; wide HAs	39	13.38 ±1.49	24.33 ± 1.09	35	28.29 ± 1.07

CI—cochlear implantation; HAs—hearing aids; minimal HAs—no free-field responses or responses only up to 500 Hz in HAs; wide Has—free-field responses for at least 250, 500, and 1000 Hz in HAs; N—number of patients; 0–5—at cochlear implant activation and 5 months after CI; 9—9 months after CI.

## Supplementary Materials

The following are available online at https://www.mdpi.com/2077-0383/9/1/228/s1. Table S1: Demographic characteristics of CI patients, Figure S1: LEAQ scores at the time of cochlear implant activation in non-DFNB1 patients. (**A**) Differences in LEAQ scores in patients with minimal and wide responses provided by HAs; (**B**) Differences in LEAQ scores in patients with very early and early CI. Whiskers represent 5–95 percentile and black dots indicate outliers. Asterisks represent statistical significance, **p* < 0.05; ns, not significant, Figure S2: LEAQ scores in the fifth month after cochlear implantation in non-DFNB1 patients. (**A**) Differences in LEAQ scores in patients with minimal and wide responses provided by HAs; (**B**) Differences in LEAQ scores in patients with very early and early CI. Whiskers represent 5–95 percentile and black dots indicate outliers; ns, not significant.
