# Supplementary material for: Cochlear Implantation Outcome in Children with DFNB1 locus Pathogenic Variants
Source: J Clin Med. 2020 Jan 15;9(1):228. doi: 10.3390/jcm9010228 (PMC7019930; doi:10.3390/jcm9010228)
Supplement: Supplementary file 1 [file jcm-09-00228-s001.pdf]

Table S1. Demographic characteristics of CI patients

	**nonDFNB1 patients**	**DFNB1 patients**
Sex [M:F]	1.35 : 1 (M - 27, F - 20)	1.16 : 1 (M - 80, F - 69)
Age at CI [months]	12.04 ± 0.44 (M - 12.52 ± 0.63; F - 11.40 ± 0.63)	12.14 ± 0.23 (M - 12.14 ± 0.32; F - 12.14 ± 0.33)
CI [very early, early]	very early - 28, early - 19 (M: very early - 15, early - 12; F: very early - 13, early - 7)	very early - 83, early - 66 (M: very early - 43, early - 37; F: very early - 40, early - 29)
HAs responses [minimal, wide]	minimal - 24, wide - 23 (M: minimal - 14, wide - 13; F: minimal - 10; wide - 10)	minimal - 76, wide - 73 (M: minimal - 41, wide - 39; F: minimal - 35; wide - 34)

M—males; F—females; CI—cochlear implantation; HAs—hearing aids, minimal HAs—no free-field responses or responses only up to 500 Hz in HAs; wide HAs—free-field responses for at least 250, 500 and 1000 Hz in HAs

nonDFNB1 HL patients

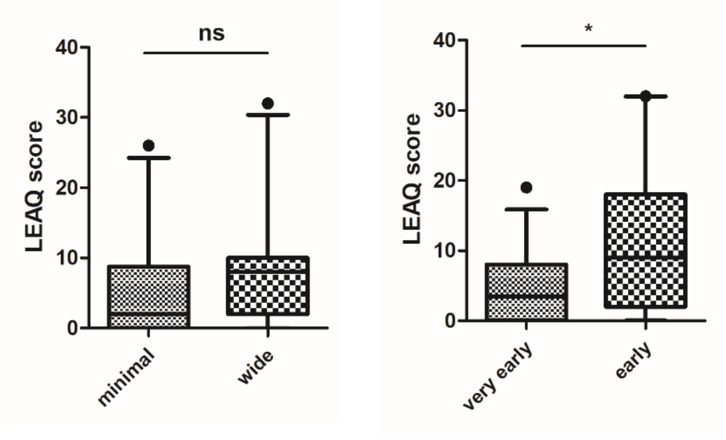

Responses with HAs (**A**)

Time at CI (**B**)

Figure S1. LEAQ scores at the time of cochlear implant activation in nonDFNB1 patients. (A) Differences in LEAQ scores in patients with minimal and wide responses provided by HAs; (B) Differences in LEAQ scores in patients with very early and early CI. Whiskers represent 5–95 percentile and black dots indicate outliers. Asterisks represent statistical significance, *$p < 0.05$; ns, not significant.

nonDFNB1 HL patients

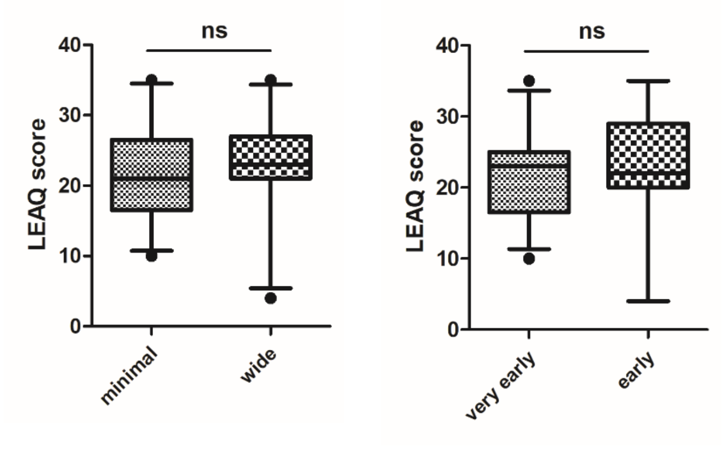

Responses with HAs
(**A**)

Time at CI
(**B**)

Figure S2. LEAQ scores at 5th month after cochlear implantation in nonDFNB1 patients. (A) Differences in LEAQ scores in patients with minimal and wide responses provided by HAs; (B) Differences in LEAQ scores in patients with very early and early CI. Whiskers represent 5–95 percentile and black dots indicate outliers; ns, not significant.
